# Impact of Automated Genotyping and Increased Breeding Oversight on Overall Mouse Breeding Colony Productivity

**DOI:** 10.3389/fphys.2022.925784

**Published:** 2022-07-18

**Authors:** Kelly R. VanDenBerg, Katherine Oravecz-Wilson, Lauren Krolikowski, Valerie Hill, Pavan Reddy, Zachary T Freeman

**Affiliations:** ^1^ Office of Research, University of Michigan Medical School, Ann Arbor, MI, United States; ^2^ Department of Internal Medicine, Division of Hematology and Oncology, University of Michigan, Ann Arbor, MI, United States; ^3^ Unit for Laboratory Animal Medicine, University of Michigan, Ann Arbor, MI, United States; ^4^ Department of Internal Medicine, Division of Hematology and Oncology, University of MI, Rogel Cancer Center, Ann Arbor, MI, United States; ^5^ Unit for Laboratory Animal Medicine, Refinement and Enrichment Advancements Laboratory, Rogel Cancer Center, University of Michigan, Ann Arbor, MI, United States

**Keywords:** breeding, genotyping, mouse model, genetically engineered (GE) animals, 3Rs (replace, reduce, refine)

## Abstract

Mice have become increasingly popular as genetic tools, facilitated by the production of advanced genetically engineered mouse models (GEMMs). GEMMs often require in-house breeding and production by research groups, which can be quite complex depending on the design of the GEMM. Identification of methods to increase the efficiency of breeding practices offers opportunities to optimize and reduce the number of animals bred for research while maintaining similar research output. We investigated the use of commercial automated genotyping and centralized breeding management on overall breeding colony productivity in a colony of multiple GEMM lines. This study involved a three-group study design, where the first group continued their standard breeding practices (group A), the second utilized standard breeding practices but outsourced genotyping in place of inhouse genotyping (group B), and a third group outsourced genotyping and had assistance with routine breeding practices from the laboratory animal care team (group C). Compared to standard practice (group A), groups B and C produced more cages and mice over time, which appeared to be driven primarily by an increase in the number of breeding cages in each colony. Higher numbers of breeders correlated with an increased number of litters and generation of new cages. The increases in colony productivity measures were further enhanced in group C compared to group B. The overall cost associated with producing new animals was lowest in group B, followed by groups A and C. Although, by the end of the study, cost to produce new mice was comparable between all three groups. These data suggest that by optimizing breeding practices and management, fewer animals could be utilized to produce the same amount of progeny and reduce overall animal usage and production.

## Introduction

Scientific and medical advancements rely on mechanistic, safety, and efficacy data acquired from various means and often include animal testing. The 3Rs, replace, reduce, and refine, serve as guiding principles to encourage continual improvement in the way animals are used for these purposes (Russell and Burch, 1959). Rodents are a popular animal model, in part, due to their high degree of genomic similarity to humans and relatively short reproductive cycles, allowing for production of large amounts of progeny in a short time (Anderson et al., 2015). The advent of genetically engineered mouse models (GEMM) coupled with CRISPR have accelerated the utilization of rodent models in biomedical research (Mou et al., 2015; Lee et al., 2020). Accompanying this increase are more demands on resources associated with colony maintenance, requirements for optimizing animal usage, and opportunities to rely on the guiding principles of the 3Rs (Finlay et al., 2015).

Breeding rodents efficiently is impacted by many factors, and it can be challenging to determine in advance how many animals to produce for ongoing research while limiting production of excess animals (Hampshire and Davis, 2005). Identifying methods that help improve the efficient management of mouse colonies and optimize animal production is pivotal for meeting experimental demand within the 3Rs framework. Since many factors can impact the overall breeding performance of a rodent colony (Hampshire and Davis, 2005), breeding may need to be optimized by mouse strains and GEMMs to enhance overall colony performance (Conner, 2002). Many mouse strains and GEMM lines can have differences in overall reproductive performance that may differ from the baseline strain characteristics (Linder, 2003). Additionally, mice breeders are known to produce consistently for the first five litters, at which point they decrease in breeding efficiency (Finn, 1963; Allen et al., 2021). Hence, length of time breeders are maintained is one of many factors that can impact colony performance.

Assessment of genotypic alterations in GEMMs is critical to ensure the findings from their use is reproducible and a judicious use of animals is practiced. Depending on the specific construction of GEMMs, an incomplete understanding of the genetic alterations can impact overall research findings (Eisener-Dorman et al., 2009; Goodwin et al., 2019; Sailer et al., 2021). Pup genotyping prior to further breeding or research activities is a critical component required to preserve the genetic integrity of colonies and support scientific reproducibility (Gleeson et al., 2021). Many methods exist for genotyping mice and take time to perform, typically with batching which allows for optimal sample processing. Delays in genotyping, or acting on results from genotyping, contribute to excess cage utilization and reduced research productivity. Robotic-assisted automated genotyping methods allow for standardization of methods and address some of these concerns (Linask and Lo, 2005). In recent years, automated services for genotyping have become available that allow for effective genotype determination with a rapid turnaround time.

Few studies have examined how different genotyping or breeding management methods impact overall rodent breeding performance and production numbers in diverse GEMM colonies. Our goal was to determine if automated genotyping methods, alone or in conjunction with enhanced rodent colony management, would impact overall productivity in a colony containing multiple distinct GEMMs. We additionally hypothesized that utilizing automated genotyping services and increased involvement of animal care personnel in colonies would improve colony output and management. In turn, this would reduce both production time for experiments and costs associated with suboptimal colony maintenance.

## Results

**FIGURE 1:**
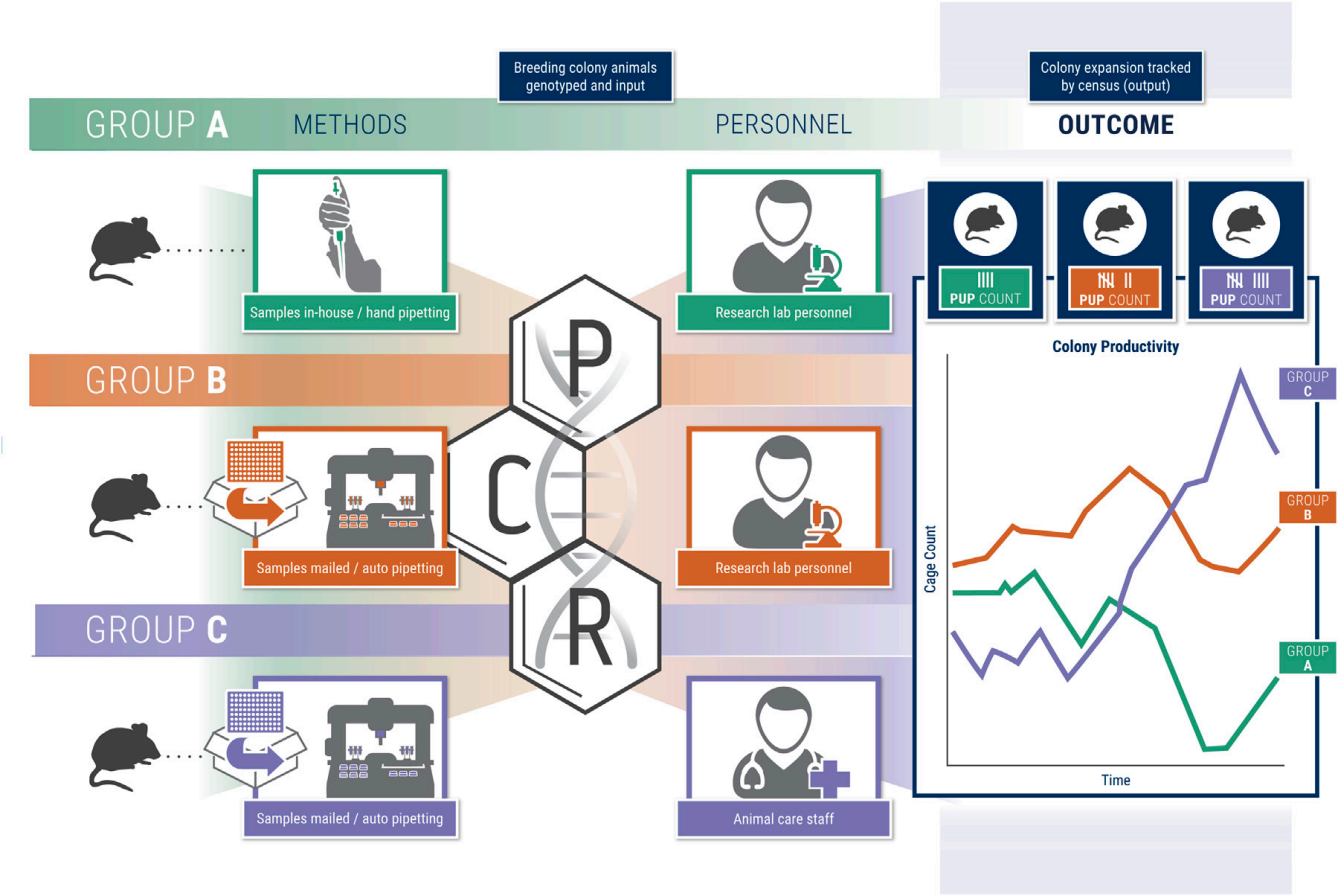
Schematic figure representing the study groups A-C and overall study design.

We compared three experimental groups to assess the effects of genotyping methods and advanced breeding management on colony output (Figure 1). Group A consisted of mice managed by the standard practice at the University of Michigan, whereby members of the investigator’s laboratory manage breeding and genotyping for each GEMM line with standard husbandry provided by the Unit for Laboratory Animal Medicine (ULAM). In group B, lab members managed breeding as in group A; however, automated genotyping services were provided by a third-party service. Group C combined identical third-party automated genotyping services as group B with enhanced ULAM management consisting of the same services provided by standard husbandry plus tag/tail, setting up breeders, resolving cages after genotyping results, euthanizing, and communicating with labs on genotyping results in real time.

**FIGURE 2:**
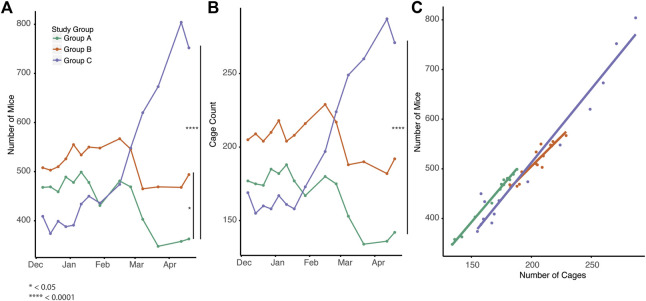
Breeding management and automated genotyping impact overall colony size. **(A)** Total number of mice for each group over the time (Mixed effect linear regression, *p* = 0.0127, 2.66e-07 for Group B and C) **(B)** Total number of cages for each group over time (Mixed effect linear regression, *p* = 6.34e-07, Group C) **(C)** Correlation of number of mice vs. number of cages (Spearman correlation r = 0.924, 0.779, 0.744, *p* = 4.461 e −08, 1.392 e −0.4, 4.021 e −04 for Group A, B, C).

First, we examined overall numbers of mice and cages across the three experimental groups. Implementation of automated genotyping without (group B) or with enhanced colony-management (group C) had a significant impact on the total number of mice compared to standard practice (group A) (Figure 2A, *p* = 0.0127, 2.66e−07 respectively). Interestingly, only group C had a significant impact on the overall number of cages over time (*p* = 6.34e−07, Figure 2B). As expected, the number of cages was significantly correlated with the number of mice across all three groups suggesting there were not differences in the ratio of mice to cages (Figure 2C, Spearman r = 0.924, 0.779, 0.744, *p* = 4.461 e −08, 1.392 e −0.4, 4.021 e −04).

**FIGURE 3:**
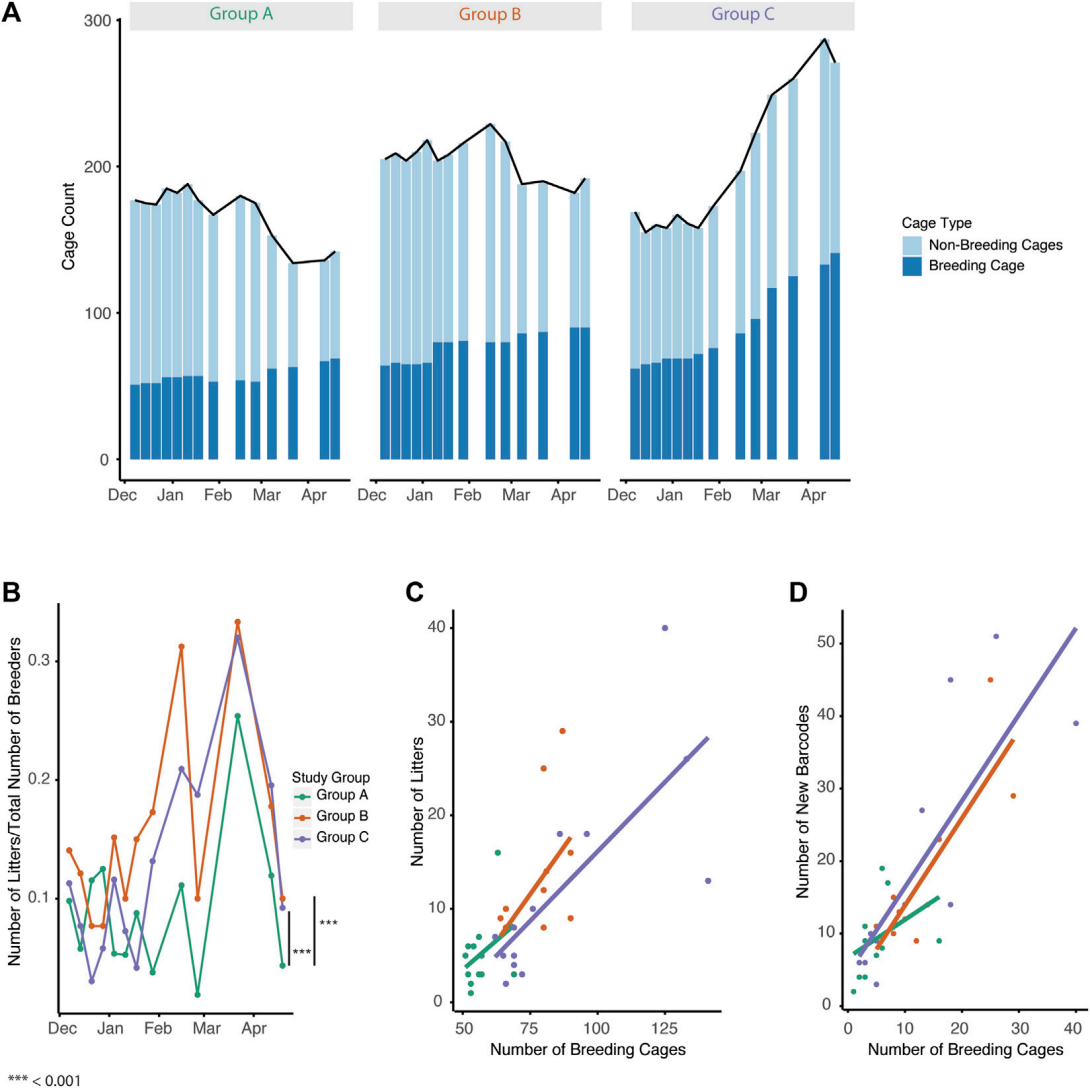
Increased breeding management and automated genotyping increased breeding efficiency. **(A)** Cage census for breeding (dark blue) and non-breeding (light blue) cages for Groups A-C. Line represents the total cage census. **(B)** Ratio of number of litters to total number of breeders for each group over time (Mixed effect linear regression, *p* = 0.00038, 0.0037 Group B, C) **(C)** Number of breeding cages compared to number of litters for Groups A-C (Spearman correlation r = 0.291, 0.622, 0.748 *p* = 0.335, 0.023, 0.0033) **(D)** Number of breeding cages compared to number of new barcodes for each group over time (Spearman correlation r = 0.051, 0.618, 0.786, *p* = 0.883, 0.0427, 0.0018)

We next examined the proportion of breeding and non-breeding cages across the three groups to determine how the overall makeup of the colonies compared. In all three groups, the number of breeding cages increased over time during the study with group C demonstrating the largest increase (Figure 3A). In groups A and B, the number of non-breeding cages decreased with time while in group C they increased. We then examined the relationship between breeding cages and the number of litters produced. Number of litters per breeder was significantly increased over time in groups B and C (Figure 3B, *p* = 0.00038, 0.0037). Interestingly, breeding cage count was correlated with litters in groups B and C but not in group A (Figure 3C, Spearman r = 0.291, 0.622, 0.748 *p* = 0.335, 0.023, 0.0033). We next compared the number of breeders with the number of new barcodes generated, as a proxy of new cages containing weaned pups, produced at each timepoint. Groups B and C had significant correlations in the number of breeding cages and the number of new barcodes produced while group A was not significant (Figure 3D, group A, B, C, Spearman r = 0.051, 0.618, 0.786 *p* = 0.883, 0.0427, 0.0018).

**FIGURE 4:**
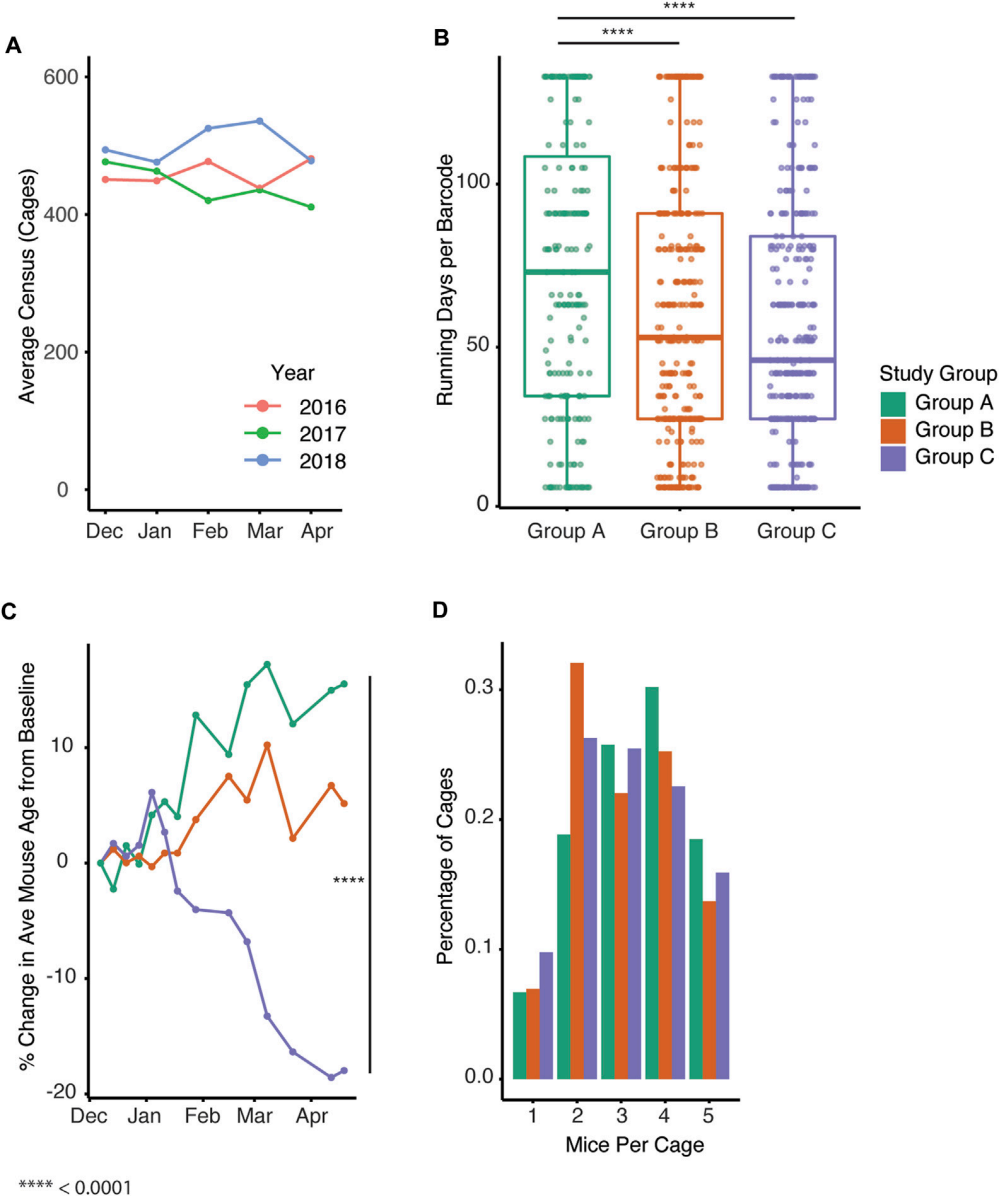
Automated genotyping is associated with increased generation of new cages and breeding performance index. **(A)** Average cage census for the study colony from December through April in past years. **(B)** Active census days per barcode for all cages in Groups A-C (*p* = 1.69 e-06, Tukey post hoc test, *p* = 4.446 e −4, 1.0 e−6, A vs. B, A vs.C) **(C)** Percent change in average mouse age from baseline over time for Groups A-C (*p* = 4.99 e−06, 0.0617. Group (B, C) **(D)** Distribution of number of mice per cage for each of the groups (Mixed effect linear regression, *p* = 1.35 e−09, 1.92 e−10, Group B, C).

Importantly, all mouse housing occurred in a consistent space footprint, whereby the space allocation for the colony was unchanged. To confirm the overall colony had a similar census despite changes in growth trends between groups, we compared census data for the past 3 years (2016–2018) to determine the colony size prior to study (Figure 4A). Given the increase in new cages generated in groups B and C within a set space allocation, we examined whether cage turnover was impacted by the different management strategies by assessing how long barcodes were in use. The date during which the barcodes were created significantly impacted the duration the barcode was active (*p* < 2 e−16). Mice group had a significant effect on the maximum number of days a barcode was active (*p* = 1.69 e−06) with group A being significantly higher than groups B and C (Figure 4B, Tukey post hoc test, *p* = 4.446 e −4, 1.0 e−6, A vs. B, A vs. C). We next examined the change in the average age of each group of mice over the study. Group C significantly impacted average age while group B approached significance (Figure 4C, *p* = 4.99 e−06, 0.0617). Automated genotyping may allow for earlier identification of a mouse’s genotype, and this could facilitate optimal housing of desired genotypes. To test this, we compared the distribution of the number of mice per cage across the three groups. The number of mice in each cage in group B and C was significantly impacted by group with the distribution being shifted towards a lower number of mice per cage (Figure 4D, *p* = 1.35 e−09, 1.92 e−10).

**FIGURE 5:**
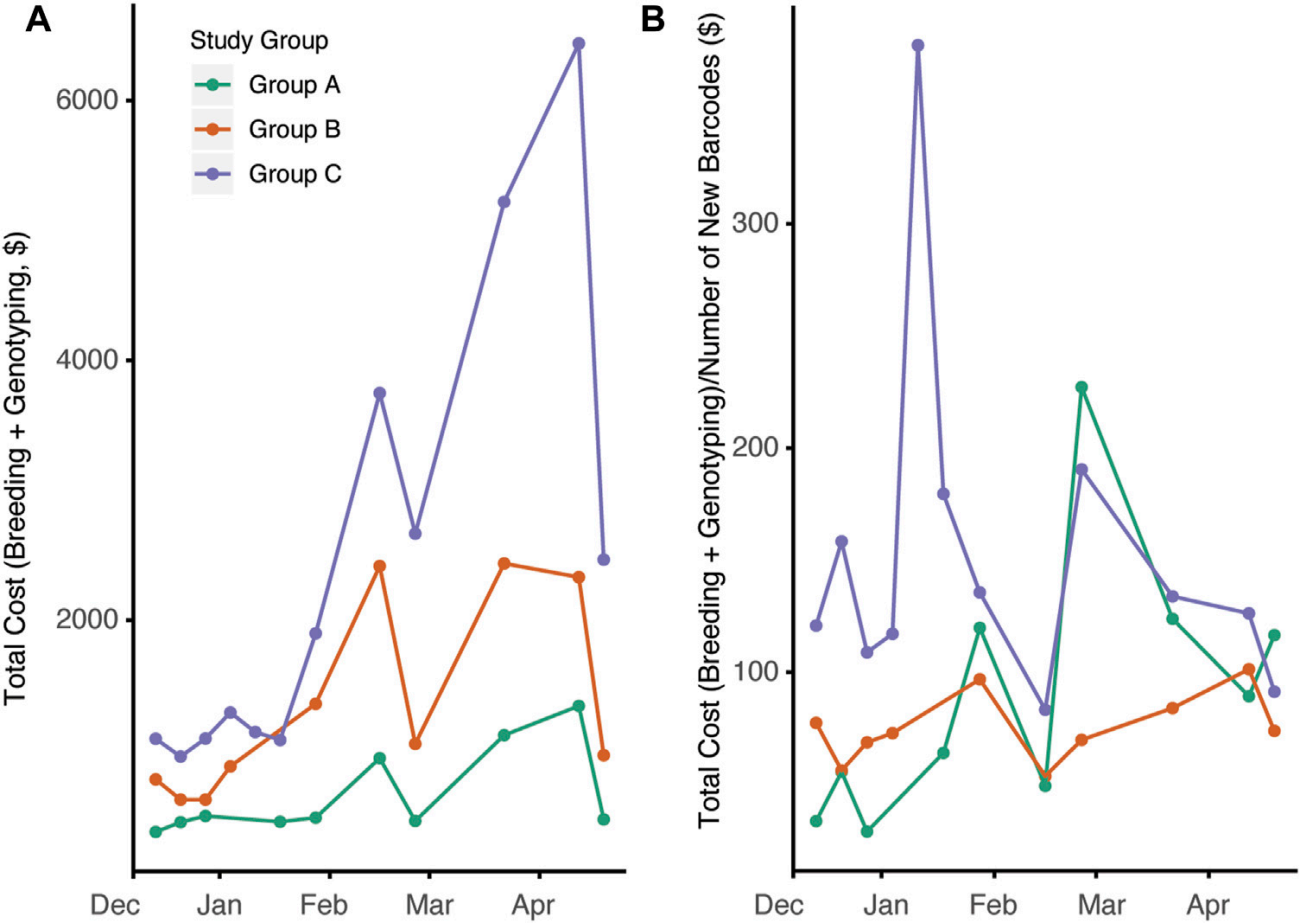
Breeding management and automated genotyping strategy impacts overall cost efficiency of mouse production. **(A)** Total cost for each day calculated consisting of total per diem costs per 2-week interval plus genotyping cost associated with new mouse production during that time frame (*p* = 0.00014) **(B)** Ratio of total costs to census new cage generated (new barcodes) over time for the three groups (*p* = 0.784,0.0135)

Changes in overall colony management are likely to have significant impacts on the cost to produce animals. The overall census cost for the duration of the study was higher in group C compared to groups A and B (Figure 5A, *p* = 0.00014). This is not surprising given the larger number of cages in group C and the increased per diem rate associated with the increased breeding support provided. Since automated genotyping is associated with increased costs, we next compared the total cost of per diems and genotyping with the total number of new barcodes generated (Figure 5B, *p* = 0.784,0.0135). When observing the study, group C had the highest cost/barcode; however, by later timepoints, the groups were roughly equivalent.

## Discussion

Managing GEMM rodent colonies in a resource-optimizing and responsible manner is an ever-growing necessity for research investigators (Ayadi et al., 2011). Equally important is the need to produce the fewest animals necessary to facilitate the research, consistent in keeping with the 3Rs (Elliott et al., 2018). In this study, we examined the effects that genotyping methods and husbandry can have on various aspects of mouse colony output. We found that automating genotyping through use of a third-party vendor and enhancing husbandry services led to significant improvements in overall colony productivity, compared to standard practices. Taken together, these data suggest that overall increased colony output with these methods could allow colonies with multiple GEMMs to be managed with fewer overall breeders while achieving the same colony productivity as previous methods.

The overall increases in colony output in group B and C we observed were in part attributed to having more breeders established. Number of breeders was correlated with both number of litters and new barcodes generated in groups B and C, suggesting that the breeding efficiency did not differ greatly in these groups. The productivity from new breeder cages lags after being set up by several months, thus greater differences may have been observed if these cages were followed for longer. There are several scenarios that would allow for the increase in overall number of breeders in group B and C without a change in overall available space. We saw that groups B and C generated more barcodes that were generally active for a shorter amount of time, which may be a result of more frequent cage turnover or producing more new cages at later time points. Early genotyping could also allow for recombination of cages at an earlier time point leading to fewer overall cages. In groups B and C, we observed a shift towards fewer numbers of mice per cage, which is the opposite of what we expected, potentially as a result from earlier euthanasia of non-desirable genotypes; although we could not control for multiple factors that could impact this. GEMM phenotype has the potential to impact the overall breeding performance. The GEMMs utilized in this study have varying degrees of immune alteration that may have inapparent effects on breeding that we were unable to control for. Larger studies with more distinct GEMMs would be warranted to determine this impact.

The impact of the experimental groups B and C on overall colony productivity have differing implications for researchers versus animal care programs. Individual research laboratories are often focused on maximizing colony output while at the same time minimizing the overall cost associated with maintaining the colony. Group B had similar overall colony output on a per breeder basis to group A and the higher level of overall production was largely driven by an increased number of breeders. At the same time, group B demonstrated the lowest overall cost for maintaining the colony for all groups and the lowest cost to produce each new mouse. Outsourced automated genotyping provides rapid results, requires lower input from the investigator, and ensures quicker results (Linask and Lo, 2005). In some circumstances, individuals performing genotyping may either wait to collect tail samples or store them until there are enough to process in a large batch at once that is time efficient. While this increases the efficiency of the PCR by including more samples, it decreases the efficiency of breeding and cage management by allowing cages to be housed for longer prior to obtaining genotyping results, particularly with animals of unwanted genotyping. Outsourced genotyping alleviates the need for large batching of samples, and allows for steadier submission of samples, and provides results in a timelier manner.

At a larger colony level, the impacts observed in group C have potential implications for overall colony management. Management of large GEMM rodent breeding colonies with multiple research investigators face unique challenges that differ from an individual researcher’s colonies. For example, rodents reproduce rapidly and the need for increased vivarium space can outpace existing animal space. Furthermore, decentralization of overall management of colonies can lead to inefficiencies in overall colony management that lead to less optimal space utilization (National Research Council et, 2011). The main difference in group C was that the husbandry personnel actively managed sample collection, analysis of genotype results, and decision making about results on a standard schedule. Additionally, all breeding practices in group C were managed by the husbandry personnel rather than each individual lab member, which may have led to further differences in efficiency of cage management and turn over. Coupled together, this coordination of practices across the group was associated with a further increase in total colony output and number of breeding cages compared with the other groups. An ideal control to help understand some of these questions would have been a fourth study group that utilized advanced ULAM husbandry without automated genotyping; although this was not feasible in our study based on financial and space resources. It was not feasible in our study to have separate personnel manage the breeding colonies and collect data which may have inadvertently impacted the results. Further studies may help to understand the impacts group C could have across a larger multi-investigator colony. Taken together, our findings offer potential methods that optimize utilization of existing vivarium space footprints that warrant further exploration.

Individual researchers and institutions should weigh the cost associated with these methods with the demonstrated benefits, including reduced lab effort and more efficient breeding, consistent with the 3Rs framework, to determine implementation feasibility in each individual circumstance. While cost is always a consideration when performing animal research, the benefits of 3Rs methods to improve the overall animal welfare often outweigh additional associated costs. In our study, the total cost was highest in group C while the cost to produce each animal was comparable to the other groups. In addition to the true cost to produce the mice, other factors need to be weighed that are hard to enumerate such as increased research productivity of investigative personnel given that they are not spending time genotyping.

In conclusion, our studies indicate that through utilization of third-party genotyping and enhanced animal husbandry services, colonies containing multiple GEMMs demonstrate enhanced breeding productivity. These methods potentially allow for fewer breeders to be utilized to produce similar colonies of animals, thus reducing the overall production of animals for the same research purposes.

## Materials and Methods

### Animals

All mice were housed in AAALAC-accredited animal facilities managed by Unit for Laboratory Animal Medicine (ULAM) at the University of Michigan. All studies described were reviewed and approved by the University of Michigan IACUC. Mice were evaluated for common mouse pathogens using dirty-bedding sentinels and swabbing of rack plenums throughout the duration of the study. These colonies consistently tested negative for the following excluded pathogenic agents: lymphocytic choriomeningitis virus, mouse adenovirus, *Mycoplasma* pulmonis, pinworms, Theiler murine encephalomyelitis virus, pneumonia virus of mice, reovirus, Sendai virus, mouse hepatitis virus, minute virus of mice, mouse parvovirus, epizootic diarrhea of infant mice virus, ectromelia virus, polyomavirus, and fur mites. The studies occurred starting in late fall of 2018 and continued until spring of 2019.

### Animal Housing

Mice in the study were housed in individually ventilated cages (IVC) with 75 in.^2^ of floor space (7.5 × 11.75 × 5 in., Allentown Caging, Allentown, NJ) at densities ranging from 1-5 mice per cage for nonbreeding cages. Mice were housed in socially compatible groups unless otherwise required for medical or social compatibility issues. Mice had *ad libitum* access food (5LOD or 5,008 irradiated rodent chow, LabDiet, Purina Mills International, St Louis, MO) and automated water (Ann Arbor municipal water, triple filtered). All mice were housed in two rooms within the same facility on a 12:12 -h light:dark cycle with a fixed number of racks (*n* = 9) that was consistent throughout the study. Male and female mice in breeding groups were continuously housed together in monogamous pairs with a maximum of 1 l of pups together at a time. Pups were counted at the first instance they were identifiable by husbandry care or laboratory staff. Pups were weaned by separating from the parents at 21 days of age. Standard per diems used to calculate overall costs for the timeframe of the study were $0.84 and $1.81 for regular ventilated caging and for cages managed for breeding by the ULAM husbandry team.

### Study Design

In this study, we utilized three groups of rodents within a single laboratory in which we assessed methods pertaining to breeding, husbandry, and genotyping (Figure 1). Group A consisted of rodents managed by the standard ULAM husbandry practices at the University of Michigan with the principal investigator-managing all breeding and genotyping. Group B was maintained in the same fashion as group A with the addition of utilizing a third-party service (Transnetyx) to provide genotyping. Group C also utilized identical third-party genotyping services as Group B in conjunction with enhanced ULAM husbandry services, which included the same services as standard husbandry plus tag/tail, setting up breeders, resolving cages after genotyping results, euthanizing, and following-up with labs to ensure efficient turn-around time to cage management. All three groups were represented in each of the two animal housing rooms. Available housing space for the three groups was roughly equal although this could not be fixed throughout the study. Genotyping for group A was done by the lab according to their own standards described below.

### Husbandry Information

All husbandry duties were performed by one ULAM husbandry technician for all three groups of the study. This technician performed the following duties for all study groups: standard husbandry including cage changes and weaning cages at 21 days of age. For group C only, this technician was primarily responsible for setting up new breeding cages, identifying weanling mice with ear tags, collecting tail samples for genotyping, submitting tail samples for genotyping when pups were between 14–17 days of age, interpreting results supplied from the testing vendor, euthanizing animals, and emailing lab members to determine how to maintain animals based on genotyping results. This person communicated with the laboratory using email and through the software utilized to track results. The technician was excluded from all analyses after the study. We were unable to blind the technician due to the nature of the study. To attempt to limit potential biases, a color-coded cage identification system was utilized that made it less obvious which cages belonged to each group.

## Genotyping

Tail samples that were less than 5 mm were collected from mice for genotyping at timepoints determined by the laboratory members. Samples from group B and C were shipped to the third-party service (Transnetyx, Cordova, TN) and genotyping was performed there. Group B members were provided with shipping envelopes and were responsible for collecting tail samples and submitting them for genotyping to the external vendor. Group C genotyping samples were submitted by the husbandry technician.

Inhouse genotyping was performed by the laboratory. Tail samples were incubated in DirectPCR lysis reagent (Promega, Madison, WI) with 10 mg/ml Proteinase K overnight at 55°C followed by a 60 min incubation at 85°C the following morning. For each GEMM line, 2-5 probes that have previously been validated in house for genotyping were utilized. Taq polymerase (ThermoFischer Scientific, Waltham, MA) was used to amplify the regions of interest with standard thermocycler cycle programs. After amplification and cooling, samples were run on a 1% agarose gel with Ethidium Bromide at 120V and 400 mA for 1 h with a 1 kb + ladder (Invitrogen, Waltham, MA). Images were acquired for each gel and then genotype for offspring determined.

### Strains and Randomization

A complete list of the GEMMs utilized in this study can be found in Table 1. All GEMMs were on a C57/BL6 background. GEMMs were stratified across Groups A-C to have an equal number of GEMMs per group (*n* = 36 total distinct GEMMs, *n* = 12 for each group). Group A started with 42 breeding cages (median = 3 cages/GEMM line, interquartile range = 6.75), group B with 47 breeding cages (median = 4 cages/GEMM line, interquartile range = 4), and group C with 48 breeding cages. Unique GEMMS were assigned to the three different groups based on the number of breeders in the colony while also attempting to control for the past breeding performance. Any additional new GEMMs that were acquired during the study were excluded from the study. Prior to this study, each lab member managed their own strains and we attempted to stratify individual lab members across the three groups with six lab members in group A, four lab members in group B, and four lab members in group C. Lab members and husbandry technicians could not be blinded to which study group they were in because of the need for the first group to perform genotyping. Laboratory members in the three groups did not receive any additional training on breeding, colony management, or when to collect samples for genotyping. Members in group C were able to use the breeding software (Transnetyx Colony Management Software, Cordova, TN) to track colonies as well as communicate with the ULAM technician.

**TABLE 1:** List of strains with source that were in each of the experimental groups.

Strain Name	Lab Name	Cat #	Vendor	Study Group
CD4Cre x CD4fl/fl	CD4Cre;CD4−/−		U of Michigan	A
Tg (Cd4-cre)1Cwi/BfluJ	CD4 Cre	17,336	Jackson	A
B6.mir142	mir142−/−		U of Michigan	A
Rosa26-LSL-Cas9 knockin on B6J	LSL	26,175	Jackson	A
B6.Cg-Tg (NPHS2-cre)295Lbh/J	2.5P Cre	8,025	Jackson	A
B6.XIAP^−/−^	XIAP−/−		U of Michigan	A
B6.NLRP6−/−	NLRP6−/−		U of Michigan	A
B6. cIAP1−/−	cIAP−/−		U of Michigan	A
CD4 Cre + *x* Sec23bfl/fl	C23b		U of Michigan	A
B6. CD24^−/−^	CD24−/−		Gifted	A
B6(C)-H2-Ab1bm12/KhEgJ	bm12	1,162	Jackson	A
B6. Sec23b-a	23B-A		U of Michigan	A
B6.NLRP6−/−	NLRP6−/−		U of Michigan	B
ALACre x ATG5fl/fl	ALA		U of Michigan	B
B6.129S-Atg5<tm1Myok>	ATG5fl/fl	RBRC02975	Riken	B
B6N(Cg)-Sdhaf1tm1.1(KOMP)Vlcg/2J	SdhaF1	28,474	Jackson	B
B6.Cg-Tg (Vil1-cre)997Gum/J	VillinCre	4,586	Jackson	B
B6.Sec23bfl/fl	23bfl/fl		U of Michigan	B
Vav1 Cre + *x* Sec23bfl/fl	V23b		Cross	B
CD4 Cre + *x* Sec23a fl/fl	C23A		Cross	B
B6.129P2-B2mtm1Unc/J (B2m KO)	B2M−/−	2087	Jackson	B
B6.Cg-Tg (Lck-cre)1Cwi N9 x ATG5fl/fl	LckCre;ATG5−/−	4,197	Taconic	B
Rosa26-LSL-Cas9 knockin on B6J	Cas9	26,175	Jackson	B
B6. SIIc	S11c		Cross	B
B6. GPR43^+/−^	GPR43		Deltagen	C
VillinCre + *x* ATG5−/−	VIATG5		Cross	C
2.5PCre x ATG5−/−	2.5PATG5−/−		Cross	C
B6.LCK Cre x Siglec-G fl/fl; GFP^+/+^	SLCK		Cross	C
B6.Cg-Tg (Alb-cre)21Mgn/J	ALA Cre	3,574	Jackson	C
B6.Sirt3	Sirt3−/−		Gifted	C
C57BL/6-Tg (TcraTcrb)1100Mjb/J	OTI	3,831	Jackson	C
B6. Siglec-G−/−;GFP^+/+^	Siglec−/−		Gifted	C
B6.EIIa Cre + *x* Sec22fl/fl	SEF		U of Michigan	C
Vav1Cre + *x* Sec22b	V22b		U of Michigan	C
B6.Sec22 fl/fl	Sec		U of Michigan	C
B6.Cg-Tg (Vil1-cre)1000Gum/J	Villin-Cre-1000	21,504	Jackson	C

### Data Acquisition

Census data pertaining to all three colonies was acquired using a breeding software (Transnetyx Colony Management Software, Cordova, TN) All cage cards were identified with a unique barcode that is utilized for standard billing practices within ULAM. Census was collected on the same day on a biweekly basis using an iPad (Apple Inc) to scan these cage barcodes and then record additional breeding data including demographic information about cage occupants, presence of litters, and generation of new cages. Cages from each experimental group were demarcated with a group identifier and all data collection was standardized across all three experimental groups. The colony software utilized three separate inputs for total number of mice, breeding information, and litter information to track overall productivity. Census, breeding, and litter data was exported on a biweekly basis in an excel format and stored in a cloud-based storage platform (Box.com, Redwood City, CA) until the time of data analysis. Genotyping results for group C were automatically populated into the breeding software when results were available allowing the husbandry technician to review it.

### Statistics/Data Analysis

Statistical analysis and graphical representations were performed using R version 3.4.3. Data was relatively linear and log2 transformation was utilized where relevant to further normalized distributions. Statistical approach generally used parabolic methods including linear regression and ANOVA where relevant. Mixed effect linear regression was utilized to control for repeated sampling of mice and cages over time. Within the model, random effects were utilized for the date of the data collection while fixed effects included mice group, number of breeders, number of litters, or other variables where relevant. Bar code max running days were calculated as the maximum number of days each barcode was in active use and analysis was performed using ANOVA with Tukey posttest for inter group comparisons.

Standard per diems used to calculate overall costs for the timeframe of the study were $0.84 and $1.81 for regular ventilated caging and for cages managed for breeding by the ULAM husbandry team. Assumptions were made that cost per test for in house were $5 per sample including labor and sending out cost $10 per sample. Actual cost of sending out would vary based on the number of mutated alleles and the complexity of the genotype of interest. Breeding costs per cage to produce mice at the age of weaning was calculated as the number of breeding cages* the per diem * number of days between data collection. Genotyping cost was calculated by the number of litters* average number of mice per litter for B6 mice * the cost per test. We use an average of five mouse pups per litter to determine the total cost for genotyping (Barnett et al., 1959; Wasson, 2017). Total cost to produce cages was the number of new barcodes generated divided by the sum of the breeding costs and genotyping costs.

## Data Availability

The raw data supporting the conclusions of this article will be made available by the authors, without undue reservation.
